# Evaluating the Risk of Hepatitis B, Hepatitis C, and Human Immunodeficiency Virus Among High-risk Deferred Blood Donors, Based on Deferral Reasons

**DOI:** 10.34172/aim.33374

**Published:** 2025-03-01

**Authors:** Sara Riyahi, Amir Teimourpour, Sedigheh Amini‐Kafiabad, Mahtab Maghsudlu, Zohreh Sharifi, Dariush Minai-Tehrani

**Affiliations:** ^1^Blood Transfusion Research Center, High Institute for Research and Education in Transfusion Medicine, Tehran, Iran; ^2^Biological Products and Blood Safety Research Center, High Institute for Research and Education in Transfusion Medicine, Tehran, Iran; ^3^Faculty of Biological Sciences and Technology, Shahid Beheshti University, Tehran, Iran

**Keywords:** Blood safety, Deferral reasons, Hepatitis B, Hepatitis C, Human immunodeficiency virus, Transfusion-transmissible infections

## Abstract

**Background:**

Blood donor selection is a crucial stage in reducing the risk of transfusion-transmissible infections (TTIs) and ensuring the safety of blood components. The objective of this study is to evaluate the impact of each question regarding deferral reasons on the risk of TTIs.

**Methods:**

This study was conducted in twenty blood transfusion centers between March 1, 2018, and September 30, 2019, including high-risk deferred volunteers from seven different groups. All samples from deferral volunteers were screened for HBsAg, anti-HCV, and HIVAg/Ab. Negative samples were pooled and tested for HBVDNA and HCVRNA. The results were compared with those of eligible donors. The association between high-risk behaviors and TTIs was analyzed using risk ratios and 95% confidence intervals.

**Results:**

Out of 2525 high-risk deferred volunteers, the risk of TTIs based on deferral reasons was as follows: a history of positive results of infectious tests RR: 401.6 (95% CI: 276.7‒582.8), drug abuse RR: 133.3 (61.9‒287.0), exposure to someone else’s blood RR:39.8 (5.7‒275.3), high-risk procedures RR:14.3 (7.9‒25.9), unsafe sexual behaviors RR:9.03 (4.1‒20.1), imprisonment and medical interventions RR=0.0. The deferred group had significantly higher rates of viral markers compared to eligible donors. Furthermore, one reactive HCV RNA was detected in anti-HCV negative samples.

**Conclusion:**

This study showed that deferring blood donations based on a history of positive results from infectious tests, drug abuse, unsafe sexual behaviors, exposure to someone else’s blood, and high-risk procedures is effective for ensuring blood safety. These deferral practices should be maintained. We recommend that other deferral criteria should be regularly evaluated for effectiveness.

## Introduction

 Blood transfusion is a life-saving procedure that plays a critical role in treating patients with severe blood loss and hematologic conditions. The availability, safety, and quality of blood and its components for transfusion are of utmost importance. Blood transfusion services bear the responsibility of ensuring the safety of blood donations.

 Various methods are employed to achieve the highest level of safety and reduce the risk of infectious, including the exclusion of high-risk volunteer groups (high-risk volunteer groups: Persons whose behaviors or activities are known to be associated with infectious diseases that may be transmitted by blood),^1^ promoting voluntary non-remunerated blood donations, donor education, implementing self-exclusion measures, utilizing national donor deferral registry software, implementing confidential unit exclusion, and conducting call-back, recall, and look-back procedures.^2,3^ In addition to these interventions, selection of eligible blood donors is the earliest intervention for ensuring blood safety. Conducting comprehensive health history interviews and performing physical examinations by qualified and trained physicians are also crucial steps in ensuring blood safety.^3,4^ The deferral criteria included in the health history questionnaire are categorized into two groups, aiming to enhance the safety of both the blood donor and the recipient.

 Studies have consistently demonstrated that deferring volunteers who engage in high-risk behaviors for blood-borne infections by implementing rigorous health history questionnaires and conducting thorough physical examinations is a critical measure for ensuring the safety of blood components and protecting blood recipients.^5-9^ An effective approach to evaluate this is by screening deferred individuals using blood-borne infection tests and comparing their positivity rates with those of eligible donors.^3^

 However, the available studies investigating the association between deferral due to high-risk behaviors and screening tests are relatively limited. One notable study conducted in Iran by Razjou et al revealed a higher prevalence of hepatitis B virus (HBV), hepatitis C virus (HCV), and human immunodeficiency virus (HIV) among deferred volunteers compared to eligible donors.^7^

 In a study conducted by Zou et al, the American Red Cross (ARC), the prevalence of viral infections among blood volunteers deferred due to potential risk was investigated. The findings revealed that deferred volunteers with a risk of viral hepatitis and a history of intravenous drug abuse had a higher prevalence of viral hepatitis compared to those who were not deferred.^10^ Similarly, a study conducted in Brazil demonstrated that deferred blood donors with high-risk behaviors had a significantly higher prevalence of HIV and syphilis compared to first-time eligible donors.^11^

 To ensure the safety of blood and its components, and considering the changing patterns of high-risk behaviors in society, it is essential to continuously evaluate and revise the questionnaire as needed.

 Considering that in Iran, only the prevalence of transfusion-transmissible infections (TTIs) in deferral individuals has been studied without considering the reason for deferral, and studies such as the one conducted by Zou et al evaluated risk factors, therefore to further investigate the impact of the donor history questionnaire, specifically the questions related to high-risk behavior, we conducted a study to recruit deferred blood volunteers with high-risk factors.

 The main objective is to assess the risk of HIV, HCV, and HBV among high-risk deferred volunteers based on deferral categories. In our previous study, we compared the prevalence of TTIs among high-risk deferred donors and eligible donors.^5^

## Materials and Methods

###  Study Design and Setting

 This cross-sectional study was conducted over 1.5 years, from March 1, 2018, to September 30, 2019, in 20 out of 91 blood transfusion centers of the Iranian Blood Transfusion Organization (IBTO). These centers account for approximately 50% of all blood donations nationwide. In this study, we chose these centers based on the IBTO experts’ opinion and experience. These centers spread geographically all over the country, from north to south and east to west in different provinces.

###  Participant Selection and Data Collection

 Pre-donation interviews were conducted in a private setting by qualified physicians for each blood donor volunteer. These interviews adhered to the established IBTO standard operating procedures (SOPs). Based on the SOPs, the physician determined the suitability of each volunteer for blood donation. If any threats for either the donor or blood recipient were detected, the physician had the authority to defer the blood donation for the donor. We included volunteers who were deferred during the interview process for TTI-related reasons and later agreed to participate in the study. Those deferred for any other reasons, or those who failed to donate, were excluded. The deferred volunteers who were included in the study were added sequentially. Demographic characteristics including sex, age, education level, marital status, donation history (first-time, repeated, and regular donor) along the reasons for deferral were considered as independent variables. Since the reasons for deferral have a high variety, we categorized them into seven categories based on the similarity of the reasons for deferral (Supplementary file 1, Table S1).

 A blood sample was collected from each selected individual for viral screening tests to determine the TTI status and used as the outcome of the study. Table S1 reports the frequency of deferral category in terms of reasons and TTI disease. The sample size of the study was estimated based on information from a previous study and the primary objective of our study.^5^

 The control group in this study consisted of eligible donors who passed the interview and physical examination and successfully donated blood, in the same period and blood transfusion centers. Required information including the frequency of total eligible donors along with the frequency of TTI among them was extracted from the IBTO database.

###  Laboratory Methods

 The enrolled deferred volunteers, as well as the eligible donors, were screened for three viral markers: HBsAg (Monalisa HBs Ag ULTRA, Bio-Rad France), HCV Ab (Monalisa Anti-HCV PLUS Version 3, Bio-Rad France), and HIV Ag/Ab (Genscreen ULTRA HIV Ag/Ab, Bio-Rad France), following the instructions provided by the respective test kits. Initially, reactive samples underwent duplicate retesting. All samples that tested reactive for HBsAg, HCV Ab, and HIV Ag/Ab were further confirmed using Hepatitis B core antibody (Enzygnost Anti-HBc monoclonal, Siemens Germany), HBsAg confirmatory (Enzygnost HBsAg Confirm, Siemens Germany), HCV Blot (INNO-LIA HCV Score, Fujirebio Europe NV), and HIV Western Blots (INNO-LIA HIV Ⅰ/Ⅱ Score, Fujirebio Europe NV).

 In the deferred group, every 10 negative samples for HBsAg, anti-HCV, and HIV Ag/Ab were pooled. The genome was then extracted using the QIAamp DNA and RNA Blood Mini-Kits (Qiagen – Germany) following the manufacturer’s procedure. The extracted samples were amplified using in-house NAT kits. To serve as an internal control, a plasmid containing a fragment of Bromo Mosaic Virus (BMV) was utilized.

 The sensitivity of the test kit was 300 IU/mL for HBV DNA, and 380 IU/mL for HCV RNA in IBTO.^12^

###  Statistical Analysis

 The frequency and percentage of HBV, HCV, and HIV in terms of deferral categories and demographic characteristics were determined. The chi-square test was used to assess the association between two categorical variables and if needed, Fisher exact tests were used. Risk ratios (RRs) along with associated 95% confidence intervals (CIs) were reported as effect size. Statistical significance was set at 0.05. All statistical analyses were conducted in the R software version 4.2.0. The epitools package was used to compute the risk ratio and its 95% confidence interval.

## Results

 A total of 2525 high-risk deferred volunteers were included in the study. Most of the study population were males (85.9%) and in the 25‒34-year age group (41.9%). The reasons for deferral in decreasing order of frequency were a history of high-risk procedures (46.0%), unsafe sexual contact (39.7%), medical interventions (6.0%), history of positive results of infectious tests (3.1%), drug abuse (2.7%), exposure to someone else’s blood (1.5%), and imprisonment (1.0%). Significant associations were observed between the category of deferral and volunteers’ characteristics (Table 1).

**Table 1:** Frequency Distribution of Each Category of Deferral by Demographic Characteristics

**Characteristics**	**Category of Deferral**							**Total**	***P *** Value*
	**Exposure to Someone else’s blood (%)**	**Unsafe Sexual Contact (%)**	**High-risk Procedures** ** (%)**	**Imprisonment** ** (%)**	**Medical Interventions** ** (%)**	**History of Positive Results of Infectious Tests (TTI) (%)**	**Drug Abuse** ** (%)**		
Sex									
Male	28 (1.3)	960 (44.3)	889 (41)	24 (1.1)	128 (5.9)	75 (3.5)	65 (3.0)	2169 (100)	< 0.001
Female	10 (2.8)	43 (12.1)	272 (76.4)	0 (0.0)	24 (6.7)	4 (1.1)	3 (0.8)	356 (100)	
Age (y)									
< 25	7 (1.2)	372 (63.4)	186 (31.7)	2 (0.3)	7 (1.2)	7 (1.2)	6 (1.0)	587 (100)	< 0.001
25‒34	16 (1.5)	488 (46.2)	444 (42.0)	12 (1.1)	46 (4.4)	22 (2.1)	29 (2.7)	1057 (100)	
35‒44	8 (1.6)	113 (23.0)	288 (58.5)	2 (0.4)	36 (7.3)	24 (4.9)	21 (4.3)	492 (100)	
45‒54	7 (2.3)	22 (7.3)	195 (64.6)	6 (2.0)	47 (15.6)	16 (5.3)	9 (3.0)	302 (100)	
55‒65	0 (0.0)	8 (9.2)	48 (55.2)	2 (2.3)	16 (18.4)	10 (11.5)	3 (3.4)	87 (100)	
Donation history									
First-time	18 (1.3)	634 (47.0)	573 (42.5)	12 (0.9)	37 (2.7)	23 (1.7)	52 (3.9)	1349 (100)	< 0.001
Repeat	11 (2.3)	152 (31.1)	236 (48.4)	4 (0.8)	66 (13.5)	17 (3.5)	2 (0.4)	488 (100)	
Regular	9 (1.3)	217 (31.5)	352 (51.2)	8 (1.2)	49 (7.1)	39 (5.7)	14 (2.0)	688 (100)	
Education									
Less than diploma	10 (1.5)	200 (30.1)	340 (51.2)	15 (2.3)	36 (5.4)	25 (3.8)	38 (5.7)	664 (100)	< 0.001
Diploma	14 (1.2)	515 (43.0)	539 (45.0)	7 (0.6)	60 (5.0)	38 (3.2)	26 (2.2)	1199 (100)	
University degree	14 (2.1)	288 (43.5)	282 (42.6)	2 (0.3)	56 (8.5)	16 (2.4)	4 (0.6)	662 (100)	
Marital status									
Single	15 (1.2)	842 (67.1)	327 (26.1)	10 (0.8)	24 (1.9)	14 (1.1)	23 (1.8)	1255 (100)	< 0.001
Married	23 (1.9)	140 (11.5)	812 (66.5)	13 (1.1)	126 (10.3)	63 (5.2)	44 (3.6)	1221 (100)	
Divorced/widow	0 (0.0)	21 (42.9)	22 (44.9)	1 (2.0)	2 (4.1)	2 (4.1)	1 (2.0)	49 (100)	
Total	38 (1.5)	1,003 (39.7)	1,161 (46.0)	24 (1.0)	152 (6.0)	79 (3.1)	68 (2.7)	2,525	-

*Comparing the association between deferral volunteers’ characteristics with the category of deferral.

 Regarding the association between the risk of TTIs among deferred donors and eligible donors, we observed that the risk of TTIs in the deferred group was 26.9 times (RR: 26.9; 95% CI: 20.0‒36.2, *P* < 0.001) higher than that of eligible donors. Similar results were observed for HIV (RR:33.3; 95% CI:10.4‒106.8; P < 0.001), HBV (RR: 26.0; 95% CI: 17.9‒37.7, *P* < 0.001), and HCV (RR:27.9; 95% CI: 15.9‒48.6; *P* < 0.001). Regarding the seven different categories of deferrals, we observed a significantly higher risk of TTIs for expose to someone else’s blood, unsafe sexual behaviors, high-risk procedures, history of positive infectious tests, and drug abuse (Table 2). Neither the imprisonment nor medical interventions categories had any positive cases. Table 2 represents the frequency, prevalence (per 100 000), and risk ratio of TTIs in terms of different deferral categories.

**Table 2:** Risk of TTIs Among Deferral Categories in Comparison with Eligible Donors

**Eligible Donors**		**N**		**HIV**	**HBV**	**HCV**	**TTIs**
		**1315871**	***n*** ** (Per×10**^5^**)**	**47 (3.6)**	**581 (44.2)**	**243 (18.5)**	**871 (66.2)**
Deferral categories	Expose to someone else’s blood	38	n (Per × 10^5^)	0 (0)	0 (0)	1 (2632)	1 (2632)
			RR	—	—	142.5	39.8
			95% CI	—	—	20.5-989.7	5.7-275.3
			*P *value	—	—	0.007	0.025
	Unsafe sexual behaviors	1,003	n (Per × 10^5^)	1 (99.7)	2 (199)	3 (299)	6 (598)
			RR	27.9	4.5	16.2	9.03
			95% CI	3.86-202.1	0.0-11.5	5.2-50.5	4.1‒20.1
			*P *value	0.036	0.074	< 0.001	< 0.001
	High-risk procedures	1,161	n (Per × 10^5^)	0 (0)	8 (689)	3 (258)	11 (947)
			RR	—	15.6	13.9	14.3
			95% CI	—	7.8-31.3	4.5-43.6	7.9‒25.9
			*P *value	—	< 0.001	0.001	< 0.001
	Imprisonment	24	n (Per × 10^5^)	0 (0)	0 (0)	0 (0)	0 (0)
			RR	—	—	—	—
			95% CI	—	—	—	—
			*P *value	—	—	—	—
	Medical interventions	152	n (Per × 10^5^)	0 (0)	0 (0)	0 (0)	0 (0)
			RR	—	—	—	—
			95% CI	—	—	—	—
			*P *value	—	—	—	—
	History of positive results of infectious tests	79	n (Per × 10^5^)	2 (2532)	18 (22785)	1 (1266)	21 (26582)
			RR	708.8	516.1	68.5	401.6
			95% CI	175.2-2867.9	341.1-780.7	9.7-482.5	276.7‒582.8
			*P *value	< 0.001	< 0.001	0.015	< 0.001
	Drug abuse	68	n (Per × 10^5^)	0 (0)	1 (1471)	5 (7353)	6 (8823)
			RR	—	33.3	398.2	133.3
			95% CI	—	4.7-233.4	169.7-934.4	61.9‒287.0
			*P *value	—	0.029	< 0.001	< 0.001

*95% CI based on bootstrap.

 In addition to the categories of deferral, the risk of TTIs among some important and selected reasons for deferral are evaluated and reported in Table 3. In Tables 2 and 3, the results of comparing risk of TTIs in terms of deferral categories and selected deferral reasons are reported.

**Table 3:** Risk of TTIs Among Selected Deferral Reasons, in Comparison with Eligible Donors

**Eligible Donors**	**N**		**HIV**	**HBV**	**HCV**	**TTIs**
	**1,315,871**	***n*** ** (Per×10**^5^**)**	**47 (3.6)**	**581 (44.2)**	**243 (18.5)**	**871 (66.2)**
Sexual contact with the HIV-positive partner	14	n (Per × 10^5^)	1 (7142.9)	0 (0.0)	0 (0.0)	1 (8333.3)
		RR	1999.8	—	—	107.9
		95% CI	296.1-13507.3	—	—	16.3-714.2
		*P *value	< 0.001	—	—	0.009
Extramarital sexual activity	792	n (Per × 10^5^)	0 (0.0)	2 (252.5)	2 (252.5)	4 (505.1)
		RR	—	5.72	13.7	7.6
		95% CI	—	1.4‒22.9 0.048	3.4‒54.9 0.009	2.9‒20.3 0.002
		*P *value	—			
Sexual contact for money or drugs	143	n (Per × 10^5^)	0 (0.0)	0 (0.0)	1 (699.3)	1 (699.3)
		RR	—	—	37.9	10.6
		95% CI	—	—	5.3‒268.1	0.01‒33.3
		*P *value	—	—	0.026	0.090
Wet cupping (*Hijama*)	903	n (Per × 10^5^)	0 (0)	8 (885.9)	2 (221.5)	10 (1107.4)
		RR	—	20.1	12.0	16.7
		95% CI	—	10.0‒40.2	2.9‒48.2	9.0‒31.1
		*P *value	—	< 0.001	0.013	< 0.001
Acupuncture	26	n (Per × 10^5^)	0 (0)	0 (0)	1 (3846.1)	1 (3846.1)
		RR	—	—	208.3	58.1
		95% CI	—	—	30.4‒1429.2	8.5‒397.5
		*P *value	—	—	0.005	0.017
History of inhalation drug use	28	n (Per × 10^5^)	0 (0)	0 (0)	1 (3571.4)	1 (3571.4)
		RR	—	—	193.4	53.9
		95% CI	—	—	28.1‒1330.7	7.9‒370.2
		*P *value	—	—	0.005	0.018
History of injection drug use	40	n (Per × 10^5^)	0 (0)	1 (2500)	4 (10000)	5 (12500)
		RR	—	56.6	541.5	188.8
		95% CI	—	8.2‒392.8	211.9‒1383.7	82.9‒429.9
		*P *value	—	0.018	< 0.001	< 0.001

*95% CI based on bootstrap. N, Total number of the study population n, Number of the study population who were infected with HIV, HCV, and HBsAg at each level of deferral reasons.

 We observed significantly higher risks of TTIs among deferred donors with sexual contact with an HIV-positive partner, extramarital sexual activity, wet cupping, acupuncture, history of inhalation drug use, and history of injection drug use.

###  HCV RNA and HBV DNA Test

 All HBsAg-negative samples tested by the nucleic acid test showed negative results for HBV DNA.

 HCV RNA was tested in the anti-HCV negative samples using mini pooled NAT. One sample, belonging to a married man who was a first-time blood donor and a drug abuser, was found to be reactive.

## Discussion

 Results from the present study indicate that volunteers who were deferred due to high-risk behaviors, including high-risk procedures for HBV and HCV, unsafe sexual contact for HIV and HCV, drug abuse for HBV and HCV, history of blood exposure for HCV, and history of positive results of infectious tests for HBV, HIV, and HCV, were more likely to have higher viral marker risk compared to eligible donors (*P *value < 0.05).

 None of the enrolled volunteers who were deferred for two categories of deferral, namely imprisonment and medical interventions, tested positive for any viral markers. However, it should be noted that in the case of deferral due to imprisonment history, despite the implementation of preventive programs and efforts to improve the health of the incarcerated population, historical studies suggest that the prevalence of HBV and HCV in prisons tends to be higher than the general population.^13-16^

 In the case of deferral due to a history of medical interventions within 12 months before the interview, while the decontamination of endoscopic and dental equipment has been improved, further information is required to assess blood safety and endoscopy and dental care.^17^

 The primary reason for deferral among volunteers testing positive for HBV is a history of positive HBV test results, primarily occurring in individuals aged 35 to 44 years. This finding could be attributed to the implementation of HBV vaccination in Iran for infants since 1993 and adolescents since 2007.^18,19^

 It appears that with the advancements in safety measures during medical interventions and improved screening for anti-HCV in blood donations since 1996,^20^ injecting drug abuse has emerged as the most significant risk factor for hepatitis C in comparing with blood donors. Consistent with other studies conducted in Iran, the findings of this study also confirmed a high prevalence of HCV among individuals with a history of injecting drug abuse.^15,21^ Furthermore, a study published by Zou et al in 2006 reported a higher prevalence of HCV among deferred individuals with a history of intravenous drug abuse compared to non-deferred donors.^10^ Moreover, high-risk volunteers may donate blood to assess their infection status after engaging in high-risk behaviors, such as injection drug use. It is recommended to conduct a thorough examination of the injection site. Wet cupping (*Hijama*) therapy is a widely practiced traditional approach to medicine in certain parts of the world. However, the practice of wet cupping carries the risk of viral infections, including Hepatitis B and C.^22^ In the present study, the primary reason for deferral, encompassing both men and women, was attributed to wet cupping. Additionally, among Hepatitis B and C patients, 10 cases reported a history of wet cupping. Notably, wet cupping accounted for eight cases of positive HBV results in this study, making it the second most common deferral reason after a history of reactive tests for HBV. This risk of infections associated with wet cupping was significantly higher than that observed among high-risk volunteers with other reasons for deferral.

 The deferral rate attributed to unsafe sexual activity may indicate the successful development of trust between the blood center and the volunteers during the interview process. However, the relatively low risk of positive cases for TTIs in comparison with eligible donors could be attributed to the increasing level of public awareness regarding the transmission of these sexually transmitted diseases.

 In today’s context, tattooing has become a common cosmetic procedure, particularly among women. The present study revealed that tattooing ranks third among the causes of high-risk deferral, following wet cupping and extramarital sexual activity. It is recommended to conduct a study that investigates the associations between tattooing and the acquisition of TTIs.

 The implementation of molecular methods has significantly enhanced the safety of blood components by reducing the pre-seroconversion window period and lowering the risk of transfusion-transmitted infections.^23-25^ Identification of an HCV-positive result through molecular testing in deferred volunteers with a negative serologic result validates the impact of the donor selection process.

## Limitations

 To determine the individual impact of factors such as endoscopy, dental care, or similar interventions, as well as imprisonment lasting more than 72 hours, on blood safety, a more comprehensive analysis is necessary. This analysis should involve a detailed examination of the risk of TTIs associated with each specific reason while ensuring an appropriate sample size. Also, due to the wide CI in some cases, it is suggested to increase the sample size in those cases in future studies.

 It is also recommended to develop donor information materials aimed at enhancing blood donors’ knowledge and reducing the number of deferrals among volunteers.

## Conclusion

 This study demonstrated that deferrals resulting from a history of positive results of infectious tests, drug abuse, unsafe sexual behaviors, high-risk procedures, and exposure to someone else’s blood like needlestick incidents, contribute to enhancing blood safety. It is crucial to conduct ongoing and systematic evaluations to assess the impact of the questionnaire and donor selection criteria to ensure continuous improvement in blood safety, and approve them as long as they ensure appropriate exclusion of high-risk individuals. This study showed that the questionnaire separates high-risk volunteers well. On the other hand, identification of an HCV-positive result through molecular testing in deferred volunteers with a negative serologic result confirms both the fact that the use of the molecular method to detect TTIs is effective in order to reduce the pre-seroconversion window period, and the efficiency of the donor selection process in Iran.

## Supplementary Files

Supplementary file 1 contains Table S1.
