# Supplementary material for: Evaluating the Risk of Hepatitis B, Hepatitis C, and Human Immunodeficiency Virus Among High-risk Deferred Blood Donors, Based on Deferral Reasons
Source: Arch Iran Med. 2025 Mar 1;28(3):155–61. doi: 10.34172/aim.33374 (PMC12038799; doi:10.34172/aim.33374)
Supplement: Supplementary file 1 — contains Table S1. [file aim-28-155-s001.pdf]

Arch Iran Med. March 2025;28(3):155-S1

10.34172/aim.33374

Table S1. Frequency of TTIs, including HBV, HCV, and HIV, in terms of Category and Reason for deferral.

Category of Deferral	Reason of Deferral	N	HBV	HCV	HIV
History of blood transfusions, biological products, transplantation	Exposure to a person's blood or other secretions	7	0	0	0
	History of blood and product transfusions	13	0	0	0
	History of Needle Stick	16	0	1	0
	History of taking anticoagulants	1	0	0	0
	History of taking drugs derived from plasma, IVIg, Rh immunoglobulin	1	0	0	0
Unsafe sexual behaviors	History of injection drug use	1	0	0	0
	Homosexual sexual contact	25	0	0	0
	Living with a person with hepatitis B or jaundice	14	0	0	0
	Living with a spouse with hepatitis B or C or HIV	10	0	0	0
	Sexual contact for money or drugs	143	0	1	0
	Sexual contact outside the family framework	792	2	2	0
	Sexual contact with a person infected with HIV/AIDS	13	0	0	1
	Sexual contact with a person who received the drug with a shared needle	5	0	0	0
High-risk procedures	Acupuncture	26	0	1	0
	wet cupping	903	8	2	0
	Ear piercing or anywhere else on the body	6	0	0	0
	History of self-injury (*Ghamehzani*)	11	0	0	0
	tattoo	208	0	0	0
	Using a shared razor in a salon/barbershop	7	0	0	0
Imprisonment	Imprisonment lasting more than 72 hours	24	0	0	0
Medical interventions	Dental practices with the possibility of transmission of infection	12	0	0	0
	Endoscopy	140	0	0	0
History of positive results of infectious tests	A history of testing positive for HIV	18	0	0	2
	Anti HBc test positive	1	0	0	0
	History of clinical hepatitis	9	2	0	0
	History of testing positive for HBV	33	16	0	0
	History of testing positive for HCV	18	0	1	0
Drug abuse	History of inhalation drug use	28	0	1	0
	History of injection drug use	40	1	4	0
**Total**		**2,525**	**29**	**13**	**3**
